# Retraction: Recombinant *Pvs*48/45 Antigen Expressed in *E*. *coli* Generates Antibodies that Block Malaria Transmission in *Anopheles albimanus* Mosquitoes

**DOI:** 10.1371/journal.pone.0317791

**Published:** 2025-01-15

**Authors:** The PLOS ONE Editors

The *PLOS ONE* Editors retract this article [1] due to concerns about compliance with the PLOS Animal Research policy.

The article [1] states, “The protocol was approved by the Committee for Animal Ethics of MVDC.” A governmental official informed PLOS that the animal ethics committees of Malaria Vaccine and Drug Development Center (MVDC) and Fundación Centro de Primates (FUCEP) did not comply with Colombian regulations regarding the use of animals in research, and as such were not authorized to provide ethics approval for animal research. The corresponding and last authors stated that the governmental agency whom PLOS contacted does not have jurisdiction over university-approved studies.

The corresponding author provided an ethics document issued in 2004 by Universidad del Valle (UdV) for a study titled “Malaria transmission blocking vaccine: immune response and vaccine development.” Officials at UdV confirmed that the document was issued by the university’s ethics committee, but they were unable to confirm that this document related to the mouse and primate studies reported in [1]. The UdV official stated that at the time the document was issued in 2004 and when the animal studies were performed in 2013, approvals had a limited validity period of one year and there was a formal requirement for researchers to request the renewal of expired ethical approvals. They stated that they do not have a record of an application for renewal. The corresponding author acknowledged that they did not apply to the ethics committee of UdV for renewal of the ethics approval.

The corresponding and last authors stated that the protocol was resubmitted and approved by an ethics committee in 2012, soon before the experiments took place; PLOS did not receive a 2012 ethics approval document and would have concerns about FUCEP approval given the information we received from the governmental official.

CM agreed with the retraction and apologized for the issues with the published article. MAH, KR, YS, and SH did not agree with the retraction. KR stands by the article’s findings. AFV, AC, and NC either did not respond directly or could not be reached.
